# Relationship Between HDL Functional Characteristics and Cardiovascular Health and Potential Impact of Dietary Patterns: A Narrative Review

**DOI:** 10.3390/nu11061231

**Published:** 2019-05-30

**Authors:** Allison S. Bardagjy, Francene M. Steinberg

**Affiliations:** Department of Nutrition and Graduate Group in Nutritional Biology, University of California, Davis, CA 95616, USA; asteve@ucdavis.edu

**Keywords:** HDL, HDL functionality, cardiovascular, prevention, nutrition, dietary pattern, DASH, Mediterranean, prudent diet

## Abstract

Cardiovascular disease is a leading cause of death around the world. Overall diet quality and dietary behaviors are core contributors to metabolic health. While therapeutic targets have traditionally focused on levels of lipoprotein cholesterol when evaluating cardiovascular risk, current perspectives on high-density lipoprotein (HDL) have shifted to evaluating the functionality of this lipoprotein particle. Effects of diet on cardiovascular health are mediated through multiple pathways, but the impact on HDL composition and function deserves greater attention. Potential areas of investigation involve changes in particle characteristics, distribution, microRNA cargo, and other functional changes such as improvements to cholesterol efflux capacity. Various dietary patterns like the Mediterranean diet and Dietary Approaches to Stop Hypertension (DASH) diet have beneficial effects on cardiovascular health and may prevent cardiovascular events. These healthful dietary patterns tend to be rich in plant-based foods, with cardiovascular benefits likely resulting from synergistic effects of the individual dietary components. The purpose of this review is to summarize current perspectives on selected functions of HDL particles and how various dietary patterns affect cardiovascular health biomarkers, with a focus on HDL functionality.

## 1. Introduction

Cardiovascular disease (CVD) is the most common cause of mortality around the world, with approximately 17.9 million deaths attributable to CVD in 2015. Cardiovascular disease encompasses several conditions including coronary heart disease, heart failure, stroke, and hypertension [1]. A common feature of the various manifestations of CVD is atherosclerosis [1]. Atherosclerosis, the presence of fatty plaques in the walls of arteries, occurs due to the retention of modified lipoproteins in the arterial wall and subsequent maladaptive inflammatory response to the modified lipoproteins [2,3]. Atherogenesis can develop in young individuals, progress silently, and lead to clinical cardiovascular events decades later [3,4]. Early lifestyle interventions at the individual level can prevent or reduce the progression of atherosclerosis and risk of future cardiovascular events [1]. At the population level, policies and strategies to reduce CVD risk factors are especially pertinent in countries with poor health behaviors and high prevalence of obesity [4].

The American Heart Association (AHA) has defined seven core health behaviors (smoking, physical activity, diet, and body weight) and health factors (total cholesterol, blood pressure, and fasting plasma glucose) involved in cardiovascular health. An individual can be classified as having poor, intermediate, and ideal cardiovascular health depending on their health behaviors and health factors [4]. Ideal cardiovascular health is achieved by lack of clinical evidence of CVD as well as optimal levels of physiologic health factors and all health behaviors, including four to five components of a healthy diet [4]. In 2011–2012, the majority (65%) of adults in the United States met only two to four of the criteria for ideal cardiovascular health [1]. Studies have shown a strong inverse relationship between ideal cardiovascular health criteria and cardiovascular disease incidence [5,6].

Beyond the seven core health behaviors and health factors, the AHA recommends several secondary metrics for monitoring cardiovascular health. One of these secondary metrics is the presence or absence of metabolic syndrome (MetS) [4]. Metabolic syndrome is a recognized risk factor for CVD and is defined as having three or more of five key physiologic traits related to poor cardiometabolic health [1,7]. There is substantial overlap between MetS criteria and poor/intermediate cardiovascular health criteria as outlined by the AHA; however, high-density lipoprotein (HDL) is only factored into MetS criteria [1]. High-density lipoprotein plays an important role in cardiovascular health. The pool of circulating HDL in the body is comprised of a heterogeneous mixture of lipoprotein particles of varying size, density, and composition. These diverse HDL particles play crucial antiatherogenic roles in the body [8,9,10]. HDL metabolism is complex and new developments highlight the importance of HDL functionality as a focus for defining relevant biomarkers, drug targets, and effective lifestyle interventions [11]. While nutrition has long been a key component of cardiovascular health recommendations, the impact of diet quality on HDL function is less well studied.

The purpose of this narrative review is to discuss pertinent aspects of HDL functions and the role of HDL in cardiovascular health, and to summarize the effects of recommended well-characterized cardioprotective dietary patterns on CVD risk, with particular focus on HDL functionality. The methodological approach for the review was to review the literature for studies limited to humans, with no time restriction. Relevant studies included randomized controlled trials, prospective and cross-sectional studies, systematic reviews, and meta-analyses. Search terms included: high-density lipoprotein functionality, HDL, cardiovascular, diet patterns, Dietary Approaches to Stop Hypertension (DASH diet), and Mediterranean diet. 

## 2. HDL Functional Characteristics and Cardiovascular Health

Epidemiological studies have demonstrated an inverse relationship between HDL-Cholesterol (HDL-C) and risk of cardiovascular disease [12,13]. This led to the scientific premise that raising plasma levels of HDL-C would result in reduction of cardiovascular risk. Because of this relationship, pharmaceutical companies developed several drugs designed to raise HDL-C concentrations through inhibition of cholesteryl ester transfer protein (CETP) activity. Several CETP inhibitors including torcetrapib, dalcetrapib, and evacetrapib progressed to clinical trials [14]. In the case of torcetrapib, an increase in HDL-C of 72% was observed in study participants [15]. Despite this HDL-C increase, the trial was stopped due to the significant increases in cardiovascular events and total mortality [15]. Trials of dalcetrapib and evacetrapib also did not show efficacy in reduction of cardiovascular events [16].

In 2017, the REVEAL trial reported that a CETP inhibitor, anacetrapib, taken in combination with an intensive statin-based therapy increased HDL-C at the study midpoint and significantly reduced coronary events in the treatment group compared with the placebo group during the follow-up period. However, at the study midpoint, the treatment group had lower non-HDL cholesterol than the placebo group, which may have contributed to the overall reduction in coronary events observed [16]. A recent subset analysis of participants from the statin-based JUPITER trial noted an inverse relationship between baseline number of HDL particles and incident CVD. This inverse relationship was maintained after 12 months of statin therapy and was the strongest HDL-related predictor of incident CVD [17]. Human genetics studies demonstrate that extremely low and high HDL-C levels are not necessarily linked to increased risk or protection from CVD, and suggest that the HDL-C level alone is not the causal factor [18,19]. Taken together, these clinical trial results have led to the re-examination of HDL metabolism with an increased focus on the function, composition, size, and number of circulating HDL particles, rather than the amount of cholesterol carried by HDL particles, which makes up a relatively small proportion of the cargo [14,16,17]. The athero-protection associated with HDL-C depends on the functional abilities of the HDL particle itself, not on the plasma levels of HDL-C [20]. Standardized nomenclature has been proposed for HDL subfractions in order to facilitate comparisons of HDL among studies and clinical outcomes [21]. A consensus statement released by the National Lipid Association accordingly recommends that future therapeutic research into CVD risk reduction should focus on modifying HDL structure and function [15].

### 2.1. HDL Reverse Cholesterol Transport and Cholesterol Efflux Capacity

Several functional aspects of HDL particles contribute to their proposed athero-protective nature [8]. First, HDL plays a vital role in reverse cholesterol transport (RCT). Specifically, HDL mediates direct RCT in which excess cholesterol from peripheral cells, such as arterial wall macrophages or foam cells, is effluxed to HDL particles and esterified to form cholesteryl esters (CEs). High-density lipoprotein travels to the liver where the CE content is taken up by hepatic scavenger receptor B1 (SRB1) or HDL receptors and is subsequently recycled or excreted in the bile [2,12]. High-density lipoprotein particle size contributes to the efficiency of different stages of RCT. The efflux of cholesterol to HDL particles from peripheral cells via ATP-binding cassette transporter A1 is more efficient in the presence of small, dense HDL particles [8]. For binding to SRB1, larger HDL particles are more effective [8,22].

Key enzymes involved in lipoprotein remodeling and RCT are lecithin–cholesterol acyltransferase (LCAT) and CETP [23,24,25]. Lecithin–cholesterol acyltransferase, a lipoprotein-associated enzyme found primarily on HDL, esterifies cholesterol to CE. Newly formed, hydrophobic CEs migrate from the HDL particle surface to the core. Lecithin–cholesterol acyltransferase activity is considered antiatherogenic because the esterification traps CE inside the HDL core, thus limiting passive diffusion of cholesterol back to peripheral tissues and enabling transport of CEs to the liver [24].

The enzyme CETP also affects lipoprotein composition and exhibits both proatherogenic and antiatherogenic actions [23,25]. Cholesteryl ester transfer protein facilitates the transfer of CEs from the HDL core to apolipoprotein B (ApoB)-containing particles (low density lipoprotein (LDL) and very low-density lipoprotein (VLDL)) in exchange for triacyglycerol (TG). The antiatherogenic action of CETP involves transfer of CEs to LDL particles followed by hepatic uptake of LDL through the LDL receptor, a process referred to as indirect RCT [15,18]. Conversely, CETP is considered proatherogenic because it diverts CEs away from HDL, thus diminishing the amount of CE cleared by the direct RCT pathway. In addition, CE depletion and simultaneous TG enrichment of HDL particles results in formation of small, dense HDL particles that are rapidly removed from circulation by renal catabolism [23,25].

Several research methods have been developed for assessing HDL function, of which the most widely used is in vitro cholesterol efflux capacity [26]. This assay examines the first step of RCT through evaluating the capacity of a plasma specimen to induce cholesterol efflux from cholesterol-loaded macrophages such as J774A.1 or THP-1 cells. While it does not include the terminal components of the in vivo process, it has been validated as clinically relevant and has demonstrated an inverse association with the incidence of cardiovascular events in various prospective studies. Cholesterol efflux has been shown to be a predictor of CVD outcomes, independent of HDL-C levels [27,28]. Further evidence was provided by impaired cholesterol efflux capacity in CVD patients compared to controls, all of whom had high levels of HDL-C [29]. This affirms the hypothesis that HDL functionality is more important than HDL-C [30]. Cholesterol efflux capacity can be influenced by the absolute number of HDL particles, as well as particle size (small pre-B versus large HDL) and respective mechanisms involving ATP-binding cassette (ABC) transporters ABCA1 and ABCG1 or SRB1, and by other qualitative features of the particle such as lipid content, fluidity, protein cargo, and inflammatory- or oxidative-stress markers [31].

### 2.2. HDL Prevention of LDL Oxidation

High-density lipoprotein is involved in preventing LDL oxidation [8]. A feature of CVD is atherosclerotic plaque development in arterial walls. The initiating event in atherogenesis involves the presence of LDL particles in the tunica intima of the artery [2]. When LDL particles are retained in the intima, LDL-associated lipids and proteins are vulnerable to oxidation [32]. Presence of so-called oxidized LDL (oxLDL) species results in an inflammatory response in the arterial wall leading to foam cell development [2,32]. Several enzymes associated with HDL, including paraoxonase 1 (PON1) and LCAT, exhibit antioxidant behavior and may prevent LDL oxidation [8]. Specifically, PON1 hydrolyzes oxidized lipids in LDL and prevents peroxidation of LDL particles [8]. Lecithin–cholesterol acyltransferase can also hydrolyze oxidized short-chain phospholipids [8]. Like HDL-C, PON1 activity is inversely associated with CVD [33]. The protection against LDL oxidation and the removal of oxidized lipids by HDL are important antioxidant and anti-inflammatory functions of HDL. Plasma oxLDL, a circulating biomarker thought to reflect arterial wall oxLDL, has been shown to have a significant inverse relationship with HDL-C concentrations [32,34].

### 2.3. HDL Anti-Inflammatory Actions

Several functional roles of HDL are mediated by apolipoprotein A-I (apoA-I), which constitutes 70% of the total protein on HDL particles. Apolipoprotein A-I is involved in RCT, antioxidant actions of HDL, and anti-inflammatory actions of HDL [8]. A relationship between apoA-I and inflammation was noted in patients with familial hypoalphalipoproteinemia, a genetic condition characterized by very low HDL-C. In these patients, C-reactive protein (CRP) plasma concentrations were significantly elevated compared with healthy controls and inversely correlated with HDL-C and apoA-I concentration [35]. As an acute phase protein, CRP can reach peak concentrations up to 50,000-fold higher than baseline during acute inflammation [36]. C-reactive protein is also chronically elevated in low-grade inflammatory conditions like obesity [37]. Epidemiological studies report that CRP positively correlates with risk for cardiovascular events [36]. Using CRP as a predictor along with traditional risk factors can significantly improve ability to predict first cardiovascular events in patients [38].

In the general population (without a genetic predisposition to low HDL-C), decreased plasma levels of HDL and apoA-I can be induced during the inflammatory acute phase response [8]. The reduction in apoA-I during acute inflammation is caused partially by the displacement of apoA-I by serum amyloid A (SAA) on HDL particles [8]. Like CRP, SAA is an acute phase protein that is substantially elevated during acute inflammation and is also modestly elevated during chronic inflammatory conditions such as obesity, diabetes, and MetS [39]. Serum amyloid A has been shown to bind to the same region on HDL as apoA-I [40]. Mass spectrometry data suggest that amino acid residues of SAA and apoA-I form crosslinks [41]. Blockage and/or displacement of apoA-I by SAA disrupts the function of HDL particles. Specifically, SAA can bind to the proteoglycans in the arterial wall and can promote removal of HDL from circulation. These actions render HDL unable to actively participate in RCT and anti-inflammatory and anti-oxidant roles, often termed dysfunctional HDL [8,39].

### 2.4. HDL Endothelial Protection and Vasodilatant Effects

Vascular endothelial function is modulated through endothelium-derived vasorelaxing factors including nitric oxide, in homeostasis with vasocontracting factors. Attenuation of nitric oxide is a critical factor contributing to endothelial dysfunction and the development of atherosclerosis. High-density lipoprotein promotes endothelial function by inducing endothelial nitric oxide synthase (eNOS), producing nitric oxide, and thus contributing to vasorelaxation. It has been demonstrated that this occurs through two mechanisms: 1) HDL activates eNOS through binding of HDL ApoA-1 to SR-B1 in the endothelial cell caveolae, and 2) HDL lysophospholipid sphingosine-1-phospate (S1P) activates S1P receptors on endothelial cell membranes, causing downstream signaling and activation of eNOS [42,43]. Increased endothelial NO activity and responsiveness has been demonstrated acutely following HDL infusion in humans with isolated low HDL [44]. High-density lipoprotein signaling through SRB-1 independent of eNOS signaling also promotes endothelial cell migration and proliferation, which maintains the health and integrity of the vascular endothelium [45]. Collectively, this data demonstrates an important role that HDL plays in promoting endothelial health independent of RCT.

### 2.5. HDL Antithrombotic Effects

Another beneficial function of HDL is reducing blood thrombosis. This is of importance because the primary etiology of ischemic myocardial infarction and stroke is superimposed thrombus formation on a ruptured atherosclerotic plaque [46]. Supporting evidence comes from a demonstration of reduced ex vivo platelet aggregation following acute infusion of reconstituted HDL [47]. Several mechanisms contribute to this atherothrombotic protective effect, including enhanced inactivation of coagulation factor V by protein C or S, enhanced prostacyclin synthesis, downregulation of E-selectin expression, and upregulation of tissue plasminogen activator which enhances fibrinolytic properties [48,49].

### 2.6. HDL Protection Against Ischemia-Reperfusion Injury

High-density lipoprotein has been shown to decrease infarction size and resulting inflammation in animal models following ischemia-reperfusion injury [50]. This is due to a key sphingolipid component of HDL, S1P, which activates the lysophospholipid receptor, enhances NO, and inhibits inflammatory cell recruitment and cardiomyocyte apoptosis. Involvement of the S1P receptor was confirmed by use of a pharmacologic S1P agonist which reproduced the effect of reducing infarct size [51].

### 2.7. HDL Modulation of Glucose Metabolism

Another regulatory role for HDL involves actions related to glucose metabolism. Clinical demonstration of this by Drew et al. [52] showed that acute infusion of reconstituted HDL in patients with type 2 diabetes mellitus resulted in increased plasma insulin levels and reduced glucose. The most rapid effect on peripheral glucose uptake being due to a non-insulin-dependent mechanism via activation of 5′ adenosine monophosphate-activated protein kinase (AMPK) in endothelial cells, adipose tissue, and skeletal muscle [52,53]. High-density lipoprotein improves β-cell function and insulin secretion directly and reduces excess cholesterol accumulation through ABCA1 and ABCG1 transporters [54].

### 2.8. HDL-Associated MicroRNAs

In addition to the well-described protein and lipid cargo, HDL carry microRNAs (miRNAs), short sequences of double-stranded, non-coding RNA that function as post-transcriptional modifiers [55]. The miRNA include those known to be involved in gene regulation for cholesterol homeostasis, vascular adhesion molecules, and hypertension. In healthy participants with normal cholesterol levels, the five most abundant miRNAs on HDL were reported to be: miR-135a, miR-188-5p, miR-877, miR-223, and miR-760. However, dysregulation of the miRNA profile occurs in various CVD and lipid metabolism pathologies [55,56]. High-density lipoprotein participates in intercellular communications by delivering miRNA to tissues such as hepatocytes and endothelial cells, with resulting gene regulation [55,57]. It is unclear whether alterations in miRNA cargo are simply an association or indicates a biologic role for HDL-associated miRNAs in either promoting or modulating various conditions [55].

## 3. Potential Impact of Dietary Patterns on HDL Functional Characteristics

Diet is one of the seven health behaviors/factors involved in promoting cardiovascular health and is the cornerstone for lifestyle modifications. The AHA has defined five goals for a healthy diet. For the ideal cardiovascular health classification, at least four out of five of the following dietary goals based on a 2000 kcal diet must be met: include fruit and vegetable consumption (≥4.5 cups per day), fish consumption (≥2 × 3.5-oz servings per week), fiber-rich whole grain consumption (≥3 × 1-oz-equivalent serving of food with ≥1.1 g of fiber per 10 g of carbohydrate per day), sodium consumption (<1500 mg per day), and sugar-sweetened beverage consumption (≤450 kcal (36 oz) per week) [4]. The committee also recommended that these five goals be achieved within the context of a Dietary Approaches to Stop Hypertension (DASH) dietary pattern [4]. This focus on the cardiovascular health effects of overall dietary patterns rather than a single nutrient reflects current priorities in nutrition science [58]. Indeed, nutrients are not consumed in isolation. The effect of diet on cardiovascular health is the result of interactions between multiple food and beverage combinations consumed over a lifetime [59,60]. Given the large health and financial burden of cardiovascular disease worldwide, it is imperative to determine which dietary patterns offer the most cardiovascular health benefits, including looking at potential biologic effects beyond the circulating levels of lipoproteins and cholesterol [59]. The Mediterranean diet, the DASH diet, and variations of these diet patterns are associated with reduction in CVD risk factors and in some cases include favorable changes to HDL functionality [58,59,60,61]. Therefore, the remainder of this review describes current knowledge of diet patterns and components on HDL function.

### 3.1. Mediterranean Dietary Pattern

Although diets are diverse on an individual level, populations living around the Mediterranean Sea have traditionally consumed diets rich in fruits, vegetables, legumes, nuts, wheat-based cereals, fish, and especially olive oil. The Mediterranean dietary pattern is also marked by moderate consumption of poultry and dairy products, but low consumption of red and processed meat. Depending on the country, moderate consumption of wine, usually with meals, is also characteristic of the Mediterranean diet [59,62,63].

There is a consensus that the Mediterranean dietary pattern exerts some cardiovascular health benefits [58]. One of the largest and most-cited Mediterranean diet studies is the Prevencion con Dieta Mediterranea (PREDIMED) randomized controlled trial, which due to the improper randomization practices has recently been reanalyzed and republished [62]. The PREDIMED study compared the effects of three diets on prevention of cardiovascular events in individuals at risk for cardiovascular disease due to type 2 diabetes mellitus or at least three negative health factors (smoking, elevated LDL-C, low HDL-C, overweight/obesity, or family history of coronary heart disease). The three diets consisted of the Mediterranean diet supplied with extra virgin olive oil, Mediterranean diet supplied with mixed nuts, or a low-fat control diet. Thousands of participants were followed for a median of 4.8 years. The primary endpoint was defined as a composite of myocardial infarction, stroke, and death from cardiovascular causes. After adjustment, 5859 participants were included. The hazard ratio for the primary endpoint was 0.71 (95% CI 0.52, 0.97) in the Mediterranean diet supplied with the extra virgin olive oil group and 0.68 (95% CI 0.49, 0.95) in the Mediterranean diet supplied with the mixed nuts group compared with the control group with a reference hazard ratio of 1.00. This represented an approximately 30% risk reduction for both Mediterranean-type diets versus the control diet. No changes in physical activity were observed among diet groups and no differences in medications. Both Mediterranean diet groups increased olive oil, nuts, fish, and legume consumption compared with the control, strongly suggesting a protective effect of the Mediterranean dietary pattern on cardiovascular health in individuals at-risk for CVD [62].

The PREDIMED trial demonstrated that the Mediterranean dietary pattern reduces risk of CVD-related death. With those promising results in mind, investigators have attempted to elucidate the mechanisms of action of the Mediterranean diet [63]. Sub-studies of the PREDIMED and smaller, randomized controlled trials provide compelling evidence that the beneficial effects of the Mediterranean dietary pattern relate to improvements in endothelial function, inflammation, oxidative stress, HDL, or a combination of these improvements as shown in Supplementary Table S1 [61,62,64,65,66,67,68,69,70,71,72,73,74]. While these assessed outcomes are related to HDL activities, only one study by Hernaez et al. [61] has directly evaluated HDL functionality using cholesterol efflux capacity and other functional markers. With regard to the HDL-related parameters, the two longest trials have been the previously described PREDIMED and also Esposito et al. [64] who investigated the impact of consuming a Mediterranean diet versus a control diet for two years in 180 participants with MetS. Randomized controlled trials of the Mediterranean diet have shown mixed results with regard to HDL-C concentrations, with four demonstrating a significant increase, four no change, and one a significant decrease [61,64,66,68,69,70,71,73,74]. Esposito et al. [64] demonstrated that the poly- and mono-unsaturated rich Mediterranean diet increased HDL-C levels by approximately 3 mg/dL. A shift to a less atherogenic lipoprotein profile after one year in a PREDIMED sub-study was demonstrated by Damesceno et al. [70] through increases in large HDL particles as well as reductions in LDL overall and in small, dense LDL.

Endothelial function has been assessed in five trials [62,64,65,66,72], yet the only trial to indirectly assess HDL’s role in modulating endothelial function through its action on eNOS was Storniolo et al. [72]. This PREDIMED sub-study examined one-year changes in metabolites and mechanistic indicators of endothelial function, and found an increase in nitric oxide metabolites after both Mediterranean diets and a decrease in endothelin-1 (ET-1) after the diet plus nuts [72]. These serum changes were correlated with blood pressure reductions and were further upheld by alterations in gene expression, namely, upregulation of eNOS and downregulation of caveolin 2 and ET-1 receptors, suggesting these elements were all involved in vascular function regulation in response to diet.

To date, the only study to directly assess HDL function following consumption of the Mediterranean diet was conducted in a subset of 296 participants from the PREDIMED trial after one year [61]. In that subset analysis, several beneficial effects on HDL were observed. First, PON1 arylesterase activity and the HDL-induced nitric oxide production increased significantly after the Mediterranean diet with extra virgin olive oil compared with the low-fat control diet, suggesting improved HDL antioxidant capacity and stimulation of vasodilatory capacity. High-density lipoprotein cholesterol efflux capacity was significantly increased after both Mediterranean diets and the extra virgin olive oil treatment significantly increased the ability of HDL particles to esterify cholesterol and also decreased CETP activity. This suggests potential for antiatherogenic effects via an enhanced clearance of cholesterol through the RCT pathway. Consumption of the Mediterranean diet with olive oil treatment also influenced HDL particle composition. The surface phospholipid content increased and the core TG content decreased significantly after the Mediterranean diet with extra virgin olive oil versus the low-fat control diet [61]. Increased phospholipid content of HDL has been shown to increase cellular cholesterol efflux to HDL, thus potentially increasing cholesterol cleared by RCT [75,76]. Overall, this study demonstrates significant beneficial effects of a Mediterranean dietary intervention on four different aspects of HDL functionality. It is not possible to ascribe these HDL changes as causal to specific cardioprotective effects seen in the PREDIMED trial, but it suggests that changes in HDL function can be examined independent of HDL-C levels and may contribute to reducing CVD risk.

Hernaez at al. [77] have recently interrogated the associations in this study population between changes in food consumption over one year and the observed change in HDL functions and have estimated the change related to common servings of the foods. The fully adjusted multivariate linear regression models yielded significant associations of key foods in amounts achievable through diet with several HDL functions. Virgin olive oil and whole grains were independently associated with increased cholesterol efflux capacity, nuts and legumes were independently associated with increased PON1 antioxidant activity, and fish consumption was associated with increased PON1 and decreased CETP activity. This supports the limited existing data of other food interventions (virgin olive oil, lycopene-rich diet, nuts and eggs) within a healthy diet that have been shown to increase HDL functionality [78,79,80,81,82].

### 3.2. DASH Dietary Pattern

The 2015 Dietary Guidelines Advisory Committee report defined dietary patterns as “the quantities, proportions, variety or combinations of different foods and beverages in diets, and the frequency with which they are habitually consumed” [60]. A dietary pattern that consistently shows beneficial effects on cardiovascular health is the DASH dietary pattern. The DASH trial pioneered the DASH diet, which emphasizes consumption of fruits, vegetables, low-fat dairy products, whole grains, poultry, and fish in the following macronutrient ratio: 27% energy from fat, 18% from protein, and 55% from carbohydrate [83]. Red meat, sweets, and sugar-sweetened beverages are not included in the DASH diet and consumption of these items is discouraged [59,84]. Evidence for the cardiovascular benefits of the DASH dietary pattern primarily stems from two randomized, controlled-feeding studies: the original DASH trial [83] and the Optimal Macro-Nutrient Intake to Prevent Heart Disease (OmniHeart) trial [85], with additional trials examining specific food substitutions [86,87,88]. For the OmniHeart trial, 10% of energy from carbohydrates originally prescribed by the DASH diet was replaced with energy from either predominantly protein or unsaturated fat. Decreases in systolic and diastolic blood pressure were significantly greater after consuming the DASH and OmniHeart-modified DASH diets compared with their respective controls.

With the exception of the OmniHeart unsaturated fat-rich DASH diet, most DASH-based studies have shown decreases in HDL-C [85,86,89,90]. It is unclear whether these changes after consuming the DASH-based diets coincided with functional changes to HDL because functional markers of HDL were not measured. Indeed, the main outcomes of most dietary interventions involving the DASH dietary pattern have been blood pressure and blood lipids [87]. A recent umbrella review of systematic reviews and meta-analyses and a previous meta-analysis of 20 randomized controlled trials involving the DASH dietary pattern reported no significant changes to HDL-C [87,91]. Of the 20 randomized controlled trials included in the meta-analysis, fifteen trials measured HDL-C. None of these studies included a measurement of HDL functionality, such as cholesterol efflux capacity. Indeed, HDL functional assays are rarely included in clinical studies despite the important information they provide [15]. Similarly, only a few studies have reported beneficial effects of the DASH dietary pattern on HDL-related parameters such as endothelial function markers [92,93], inflammatory markers [94], and oxidative stress [95]. More research is needed to elucidate the mechanism behind the favorable changes in blood pressure and blood lipids that occur when following the DASH dietary pattern.

One study exploring macronutrient substitutions within the DASH diet did include measurement of HDL particle subfractions [88]. The randomized controlled crossover study compared consumption of three diets: a control diet, a standard DASH diet, and a higher fat, lower carbohydrate version of the standard DASH diet where full fat dairy products replaced non-fat and low-fat dairy products. Both DASH diets significantly reduced systolic and diastolic blood pressure and TC compared with the control diet. The standard DASH diet, but not the modified DASH diet, resulted in significantly lower LDL-C, HDL-C, and plasma apoA-I compared with the control. No changes in plasma apoA-I concentrations were observed for the modified DASH diet compared with the control diet. No changes in particle concentrations of small, large, or total HDL were observed between the three diets and HDL functional parameters were not assessed [88]. Future DASH-based dietary interventions should examine HDL particle characteristics and consider including measures of HDL functionality.

### 3.3. Empirically-Defined Dietary Patterns

The Mediterranean and DASH dietary patterns are well defined. An alternative approach to studying dietary patterns and CVD risk is to utilize observational dietary data to empirically define dietary patterns [96]. A recent meta-analysis investigated the relationship between CVD risk and dietary patterns defined a posteriori [97]. The researchers reviewed observational studies with empirically defined dietary patterns and reclassified the dietary patterns as prudent/healthy diet or unhealthy/western diet in order to determine the relationship with CVD risk. The prudent/healthy dietary patterns were distinguished by the presence of fruit, vegetables, whole grains, fish, and poultry, whereas the unhealthy/western dietary patterns featured processed meats, refined grains, sweets, sugary drinks, and fried foods. Five studies with total CVD risk and CVD mortality as endpoints were identified. The pooled relative risk of CVD when comparing the extremes of the unhealthy/western diet was 1.14 (95% CI 0.92, 1.42). The pooled relative risk of CVD in these studies when comparing the highest level of prudent/healthy diets with the lowest level of prudent/healthy diets was 0.69 (95% CI 0.60, 0.78) suggesting a protective effect of prudent/healthy dietary patterns [97]. Of the five studies included in the meta-analysis, two reported HDL-C. In both studies, the dietary patterns associated with the lowest incidence of CVD were also positively associated with HDL-C [98,99].

The association between food groups and intermediate CVD risk biomarkers was investigated in a systematic review and network meta-analysis [100]. Comparisons were made among food groups of refined grains, whole grains, nuts, legumes, fruits and vegetables, eggs, dairy, fish, red meat, and sugar-sweetened beverages. A secondary outcome was HDL-C and HDL functional parameters were not considered. The food groups identified as most effective at increasing HDL-C were nuts, whole grains, fish, and red meats. With the exception of the meat item, this list is consistent with the findings of Hernaez et al. [77] with regard to associations of Mediterranean diet foods and HDL functionality, including cholesterol efflux capacity and antioxidant activity.

Consumption of plant-based foods such as fruit, vegetables, nuts, legumes, and whole grains are common to the Mediterranean dietary pattern, DASH dietary pattern, and various empirically-defined prudent/healthy dietary patterns [97,99,101]. The benefits of plant-based diets on cardiovascular health and other chronic diseases are well documented and incorporation of more plant-based foods into a diet can improve diet quality [101,102]. The effects of improved diet quality and physical activity on HDL cholesterol efflux capacity were recently studied in large study involving abdominally obese, dyslipidemic, sedentary men completing a one-year lifestyle modification program [103]. Over the year-long intervention, the men in the treatment group (*n* = 113) significantly improved diet quality from baseline as measured by a DASH-derived diet quality score. Specifically, intake of fruit, vegetables, and whole grains were increased [104]. Among several favorable cardiometabolic changes, HDL-C, apoA-I, and HDL mean particle size were significantly increased from baseline in the treatment group, although HDL-C and apoA-I also significantly increased in the control group. Cholesterol efflux capacity of ApoB-depleted participant sera was measured in two cell lines. Cholesterol efflux capacity from J774A.1 macrophage cells increased from baseline by 14.1% and HepG2 hepatocellular carcinoma cell cholesterol efflux capacity increased from baseline by 3.4%. These significant increases were observed in the treatment group alone [103]. Taken together, the improved diet quality as part of a lifestyle modification that included physical activity led to improved HDL function. Teasing apart the contribution of diet quality and physical activity to HDL function or determining their synergistic effects should be investigated in future studies.

## 4. Conclusions

Effects of diet and lipoprotein metabolism on cardiovascular health are mediated through multiple pathways and the biology of HDL is complex. The beneficial effects of HDL in decreasing CVD risk are not fully reflected through the amount of cholesterol carried by the HDL (HDL-C). Rather, the quality of the HDL particle as assessed by HDL functionalities such as cholesterol efflux capacity, and indirectly through anti-inflammatory, antioxidant, and vasorelaxant properties are important parameters and hold potential for examining associations with dietary interventions. Limited data are available on the impact of diet patterns or foods and food components on HDL particle functional characteristics, indicating a need for additional research. Currently, the strongest evidence exists for the positive contributions of the Mediterranean diet to increasing HDL cholesterol efflux capacity and PON1 activity, which are associated with particular food groups. These foods also play a central part in the DASH and other healthful plant-based patterns, but limited data exists on HDL functionality after consumption of these diet paradigms. With increased knowledge of how dietary composition affects HDL functional parameters and other cardiovascular health biomarkers, recommendations for healthful dietary patterns can be made with more confidence.

## Supplementary Materials

The following are available online at https://www.mdpi.com/2072-6643/11/6/1231/s1, Table S1: HDL concentration, HDL-related Functional Parameters and Mediterranean Diet.
