# Supplementary material for: Relationship Between HDL Functional Characteristics and Cardiovascular Health and Potential Impact of Dietary Patterns: A Narrative Review
Source: Nutrients. 2019 May 30;11(6):1231. doi: 10.3390/nu11061231 (PMC6627343; doi:10.3390/nu11061231)
Supplement: Supplementary file 1 [file nutrients-11-01231-s001.pdf]

Table S1 HDL concentration, HDL-related Functional Parameters and Mediterranean Diet

Study (reference #)	Subjects N=	Dietary Intervention	Time Interval	HDL-C Concentration	HDL Profile	Endothelial Function	Cholesterol Efflux Capacity	LCAT, CTEP	HDL Antioxidant Function	Immunomodulation, Oxidative Stress Biomarker
Estruch et al. (73)	772	MED-EVOO, MED-NUT, Control	3 month	Increase in both MED diets, and vs control						Decrease in CRP with MED-EVOO diet; and in IL-6, ICAM-1, VCAM-1 in both MED-EVOO and MED-NUT
Esposito et al. (64)	180	MED, Control	2 year	Increase in MED vs control		Increase L-arginine test in MED vs control				Decrease in hsCRP, IL-6, IL-7, IL-18 in MED vs control
Davis et al. (65)	166	MED, Control	6 month			Increase FMD in MED vs control				
Davis et al. (74)	166	MED, Control	6 month	No significant change						Decrease in F2-Isoprostane MED vs control
Rallidis et al. (66)	90	Greek MED, Control	2 month	No significant change		Increase FMD in MED				
Rallidis et al. (67)	90	Greek MED, Control	2 month							Decrease in CRP, sP-Selectin, sE-Selectin in MED
Kanstantinidou et al. (68)	90	EVOO, Washed OO, Control	3 month	Decrease in EVOO						Decrease IFNγ, sP-Selectin in EVOO
Fito et al. (69)	372	MED-EVOO, MED-NUT, Control	3 month	Increase in MED-NUT						Decrease plasma oxLDL in MED-EVOO
Damesceno et al. (70)	169	MED-EVOO, MED-NUT, Control	1 year	No significant change	Increase large HDL and total particles					
Casas et al. (71)	165	MED-EVOO, MED-NUT, Control	3-5 year	Increase in both MED diets						Decrease hsCRP, IL-6, TNFα, MCP-1, and PBMC adhesion molecules CD40,

										CD49d in MED-EVOO and MED-NUTS vs Control
Storniolo et al. (72)	90	MED-EVOO, MED-NUT, Control	1 year			Increase serum NO metabolites in MED-EVOO; Decrease ET-1 in MED-NUT; PBMC gene expression altered in both MED diets			Increase serum antioxidant capacity in MED-EVOO	
Hernaez et al. (61)	296	MED-EVOO, MED-NUT, Control	1 year	No significant change	Increase large HDL in MED-EVOO and MED-NUT	Increase HDL induced NO from HUVEC cells in MED-EVOO vs control	Increase CEC from THP-1 cells in MED-EVOO	Increase LCAT and decrease CETP in MED-EVOO	Increase PON-1 and LDL lag time in MED-EVOO	

Legend: CRP = C-reactive protein; ET-1 = Endothelin-1; EVOO = Extra virgin olive oil; HDL = High-density lipoprotein; HUVEC = Human umbilical vein endothelial cells; ICAM = Intracellular cell adhesion molecule; IFN = Interferon; IL = Interleukin; MCP-1 = Monocyte chemotactic protein-1; MED = Mediterranean; NUT = Nuts; OO = Olive oil; oxLDL = oxidized LDL; PBMC = Peripheral blood mononuclear cells; PON-1 = Paraoxonase-1; THP-1 = Human monocyte cells; TNF = Tumor necrosis factor; VCAM = Vascular cell adhesion molecule
